# Oesophageal adenocarcinoma is associated with a deregulation in the MYC/MAX/MAD network

**DOI:** 10.1038/sj.bjc.6604398

**Published:** 2008-05-20

**Authors:** J K R Boult, P Tanière, M T Hallissey, M J Campbell, C Tselepis

**Affiliations:** 1 CRUK Institute for Cancer Studies, University of Birmingham, Vincent Drive, Birmingham B15 2TH, UK; 2 University Hospital Birmingham NHS Foundation Trust, Birmingham B15 2TH, UK; 3 Department of Pharmacology and Therapeutics, Roswell Park Cancer Institute, Buffalo, NY 14263, USA

**Keywords:** c-MYC, MAD, oesophageal adenocarcinoma, Barrett's metaplasia

## Abstract

Oesophageal adenocarcinoma, which arises from an acquired columnar lesion, Barrett's metaplasia, is rising in incidence more rapidly than any other cancer in the Western world. Elevated expression of c-MYC has been demonstrated in oesophageal adenocarcinoma; however, the expression of other members of the MYC/MAX/MAD network has not been addressed. The aims of this work were to characterise the expression of c-MYC, MAX and the MAD family in adenocarcinoma development and assess the effects of overexpression on cellular behaviour. mRNA expression in samples of Barrett's metaplasia and oesophageal adenocarcinoma were examined by qRT–PCR. Semi-quantitative immunohistochemistry and western blotting were used to examine cellular localisation and protein levels. Cellular proliferation and mRNA expression were determined in SEG1 cells overexpressing c-MYCER or MAD1 using a bromodeoxyuridine assay and qRT–PCR, respectively. Consistent with previous work expression of c-MYC was deregulated in oesophageal adenocarcinoma. Paradoxically, increased expression of putative c-MYC antagonists MAD1 and MXI1 was observed in tumour specimens. Overexpression of c-MYC and MAD proteins in SEG1 cells resulted in differential expression of MYC/MAX/MAD network members and reciprocal changes in proliferation. In conclusion, the expression patterns of c-MYC, MAX and the MAD family were shown to be deregulated in the oesophageal cancer model.

The incidence of oesophageal adenocarcinoma has increased more rapidly over the past three decades than any other cancer in the Western world and continues to rise (Bollschweiler *et al*, 2001; Lagergren, 2005). The prognosis for patients with oesophageal adenocarcinoma remains extremely poor with median survival time merely 18 months post-diagnosis and 5-year survival rates approximating just 10% in most European populations (Sant *et al*, 2003). Barrett's metaplasia, characterised by the replacement of the native stratified squamous epithelium of the distal oesophagus with a heterogeneous columnar mucosa, is the most prominent risk factor for oesophageal adenocarcinoma (Jankowski *et al*, 2000; Lagergren, 2005). The estimated annual risk of progression to adenocarcinoma in individuals with Barrett's metaplasia is 0.2–1%, which equates to 30–125 times the risk in the general population (Hage *et al*, 2004; Solaymani-Dodaran *et al*, 2004).

c-MYC is an oncogenic transcription factor, which, as part of a heterodimeric complex with MAX, activates the expression of a diverse range of genes implicated in cellular processes such as cell growth, proliferation, loss of differentiation and apoptosis (Nesbit *et al*, 1999; Grandori *et al*, 2000). Deregulated or elevated expression of c-MYC has been documented in a wide range of human malignancies and is often associated with tumours of an aggressive, poorly differentiated phenotype (Dang, 1999; Nesbit *et al*, 1999). The role of c-MYC in tumorigenesis is complex; its activation is mediated by a wide range of direct and indirect mechanisms (Salghetti *et al*, 1999; Bernasconi *et al*, 2000; Chiariello *et al*, 2001) and its precise role in cell proliferation, growth arrest and apoptosis is dependent on tissue type and environment (Pelengaris *et al*, 2000). In addition, myc overexpressing mice demonstrate increased incidence of tumours (Pelengaris *et al*, 1999). The expression of c-MYC in human oesophageal malignancy has previously been documented, demonstrating that c-MYC expression is elevated in Barrett's metaplasia and further overexpressed in oesophageal adenocarcinoma (Tselepis *et al*, 2003; Schmidt *et al*, 2007). The gene encoding c-MYC is located within chromosomal region 8q23–24.2, a region frequently amplified in adenocarcinomas of the oesophagus and gastro-oesophageal junction and that has been detected in Barrett's metaplasia (Walch *et al*, 2000; Croft *et al*, 2002; Doak *et al*, 2003; van Dekken *et al*, 2006).

The MAD family of transcriptional repressors, including MAD1 and MXI1, also bind to MAX and are believed to antagonise the activity of MYC proteins by competing for MAX-binding and interaction with E-box sequences in target gene promoters, and by actively repressing transcription of MYC target genes (Ayer *et al*, 1993; Zervos *et al*, 1993; Hurlin *et al*, 1995b). Their roles in terminal differentiation and growth arrest, and their ability to block MYC-induced transformation have led to them being postulated as tumour suppressor genes (Ayer *et al*, 1993; Zervos *et al*, 1993; Cultraro *et al*, 1997; Queva *et al*, 1998). However, to date little evidence to confirm this has been published and there is conflicting evidence as to whether putative MYC antagonist MXI1 is lost or mutated in human cancers (Gray *et al*, 1995; Bartsch *et al*, 1996; Kuczyk *et al*, 1998).

There have been a number of isoforms of MXI1 isolated from glioblastoma and haematological cells that suggest dominant negative activity; antagonising the normal activity of MXI1 and demonstrating differential expression (Engstrom *et al*, 2004; Kawamata *et al*, 2005). Of particular interest is MXI1-0, an isoform with an alternative first exon, which demonstrates elevated expression in glioblastoma, aberrant cellular localisation and fails to repress c-MYC dependent transcription (Engstrom *et al*, 2004).

While c-MYC expression has been widely characterised in oesophageal adenocarcinoma, expression of members of the MAD family of putative c-MYC antagonists is yet to be studied in any detail in gastrointestinal carcinogenesis. The gene-encoding MAD1 (*MXD1)* has previously been identified as one of six genes downregulated at the transcriptional level in oesophageal adenocarcinoma (Hourihan *et al*, 2003), however, this observation has not been confirmed and other members of the MAD family have not been addressed. We hypothesised that MAD expression would be repressed in the progression of oesophageal adenocarcinoma in a reciprocal pattern to c-MYC. Overexpression of MAD family proteins in other cell systems has demonstrated decreased cell cycling and reduced apoptosis, therefore, we postulate that modulating the expression of MAD1 would have a similar effect in SEG1 cells and may represent a mechanism by which tumour growth could be retarded.

## MATERIALS AND METHODS

### Ethics

This work has been carried out in accordance with the Declaration of Helsinki (2000) of the World Medical Association. Ethical approval for this study was approved by University Hospital Birmingham Trust (LREC 2003/331). All patients provided informed written consent.

### Patient tissue

**Oesophageal adenocarcinoma resection specimens.** Samples of oesophageal adenocarcinoma (*n*=37), some of which were matched with Barrett's metaplasia (BM) from the same resection specimen (*n*=11), were collected during surgery and each tissue specimen was divided for RNA and protein extraction and pathological confirmation.
**Endoscopic specimens.** Samples of long segment (⩾3 cm) Barrett's metaplasia (*n*=14), defined as columnar mucosa with intestinal type goblet cells, with matched normal oesophageal squamous mucosa (S) and gastric fundal mucosa (F) from the same patient were collected during routine endoscopy. Any patients with Barrett's metaplasia with evidence of dysplasia or adenocarcinoma were excluded from this study.
**Archived tissue.** Paraffin sections of normal oesophagus (*n*=10), normal gastric fundus (*n*=10), Barrett's metaplasia (*n*=25), Barrett's with dysplasia (BD, *n*=20) and oesophageal adenocarcinoma (OAC, *n*=25) were identified within an archived tissue bank, Department of Pathology, Queen Elizabeth Hospital Birmingham, and processed for immunohistochemistry.

### Quantitative real-time RT–PCR

Quantitative real-time RT–PCR (qRT–PCR) was performed as described previously (Brookes *et al*, 2006) using 18S ribosomal RNA as an internal standard (Applied Biosystems, Warrington, UK) and sets of primers and 5′FAM 3′TAMRA probes listed in Table 1.

### Western blotting

Western blotting was performed as described previously (Brookes *et al*, 2006) with a mouse monoclonal antibody to c-MYC (1 *μ*g ml^−1^, clone 9E10, Applied Biosystems) or a rabbit polyclonal antibody to MAD1 (1 *μ*g ml^−1^, clone C-19, Autogen Bioclear, Calne, Wiltshire, UK) or MXI1 (1 *μ*g ml^−1^, clone G-16, Autogen Bioclear). A mouse monoclonal antibody to cytokeratin 19 (CK19) (0.5 *μ*g ml^−1^, cloneA53-B/A2.26; Merck Chemicals Ltd, Nottingham, UK) was employed for normalisation of epithelial protein loading. Immunoreactive bands were subject to densitometry using a BioRad GS800 calibrated densitometer and Quantity one software. Where available a blocking peptide was used to confirm specific immunoreactive bands (MAD1 5 *μ*g ml^−1^; MXI1 5 *μ*g ml^−1^).

### Immunohistochemistry

Immunohistochemistry was performed as previously described (Brookes *et al*, 2006) using microwave antigen retrieval and antibodies to c-MYC (2 *μ*g ml^−1^), MAD1 (2 *μ*g ml^−1^, clone FL-221, Autogen Bioclear), MXI1 (2.7 *μ*g ml^−1^) or MAX (50 ng ml^−1^, clone C-17, Autogen Bioclear). Positive control tissue was included, and omission of primary antibody and, where available, blocking peptides (MXI1 13.5 *μ*g ml^−1^; MAX 250 ng ml^−1^) were used as negative controls. The slides were scored by a previously described method for (i) intensity of staining (0=negative, 1=weak, 2=moderate, 3=intense) and (ii) percentage of epithelial cells staining (0=0–5%; 1=6–25%; 2=26–50%; 3=51–75%; 4=76–100%). These two values were multiplied to yield a final staining score of between 0 and 12. In addition, cellular localisation was assessed (Di Martino *et al*, 2006). All sections were scored independently by two observers.

### Cell culture

The cell line SEG1 (Hughes *et al*, 1997; a kind gift of Dr David Beer, University of Michigan, Ann Arbour, MI) was routinely cultured in DMEM with 10% FCS supplemented with 100 U ml^–1^ penicillin and 0.1 mg ml^–1^ streptomycin (Invitrogen, Paisley, Renfrewshire, UK).

Cells were transfected with pcDNA3.1-MYCER, encoding a chimeric protein that consists of human c-MYC fused at its carboxyl terminus to the hormone-binding domain of a mutant mouse oestrogen receptor (Littlewood *et al*, 1995), pcDNA3-MAD1 or the corresponding empty vector using Lipofectamine and Plus reagent according to manufacturer's instructions (Invitrogen). Twenty-four hours after transfection with c-MYCER, the fusion protein was activated by replacing the medium with 100 nM 4-hydroxytamoxifen (4-OHT) (or control medium). Cells were cultured for a further 24 h before cells processing for RNA or protein or up to 96 h for a proliferation assay.

### Bromodeoxyuridine incorporation assay

A colorimetric cell proliferation ELISA was performed according to the manufacturer's instructions (Roche Applied Biosystems, Burgess Hill, Sussex, UK). Briefly, cells were labelled with bromodeoxyuridine (BrdU) followed by fixation and incubation with anti-BrdU peroxidase, the immune complex was then detected using a 3,3,5,5-tetramethylbenzidine substrate reaction with the reaction product assessed at 370 nm.

### Statistics

All data are presented as means±1 s.e.m. Statistical significance was calculated using paired *t*-test for mRNA analysis, Mann–Whitney test for analysis of immunohistochemical staining and unpaired Student's *t*-test for *in vitro* data. Significance was accepted at *P*⩽0.05. All analyses were performed using SPSS version 10.0 (SPSS Inc., Chicago, Illinois, USA).

## RESULTS

### mRNA expression of the MYC/MAX/MAD network in Barrett's metaplasia and oesophageal adenocarcinoma

**Figure 1:**
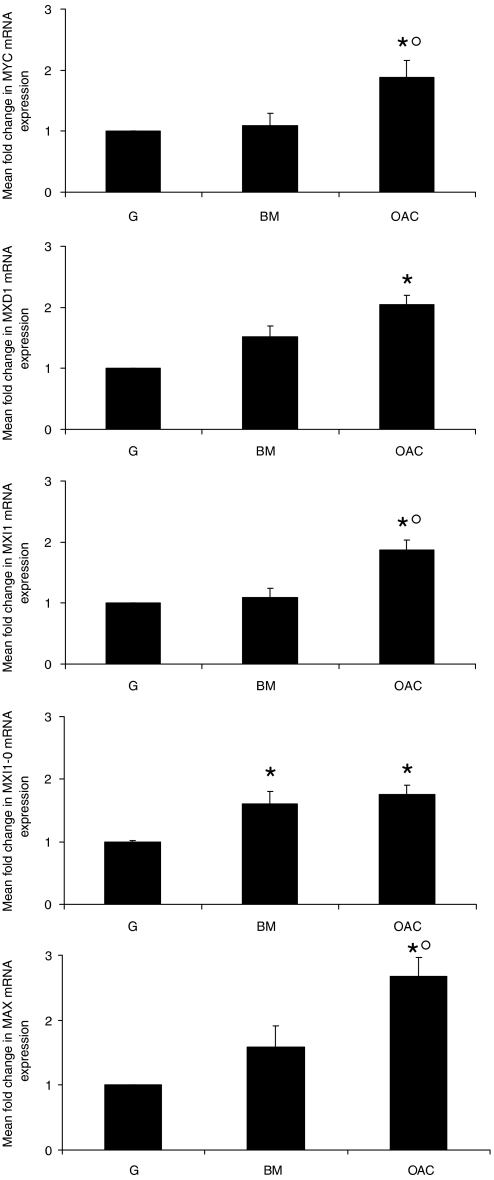
mRNA expression of MYC/MAX/MAD network genes in Barrett's metaplasia and oesophageal adenocarcinoma. qRT–PCR was used to examine expression of genes encoding c-MYC, MAD1, MXI1, MXI1-0 and MAX in Barrett's metaplasia (BM *n*=25) and oesophageal adenocarcinoma (OAC *n*=37). Graphs represent mean fold change relative to matched normal gastric control (G, normalised to one) ±1 s.e.m. ^*^ denotes significant change relative to G, ° denotes significant difference between BM and OAC (*P*<0.05).

Quantitative real-time RT–PCR was utilised to assess the expression of the mRNAs encoding c-MYC, MAD1, MXI1, MXI1-0 and MAX in normal epithelia, Barrett's metaplasia and oesophageal adenocarcinoma specimens. This revealed that all the transcripts analysed were expressed at a higher level in oesophageal adenocarcinoma tissue than in matched normal gastric (Figure 1) and oesophageal (data not shown) controls. The only gene to be significantly altered in Barrett's metaplasia in comparison with normal gastric mucosa was *MXI1-0*, the alternatively transcribed isoform of MXI1. When expression in adenocarcinoma was evaluated in comparison to matched Barrett's metaplasia it was apparent that expression of *MYC*, *MXI1* and *MAX* were significantly elevated in the malignant transformation of Barrett's metaplasia.

### Expression of MYC and MAD family proteins in Barrett's metaplasia and oesophageal adenocarcinoma

**Figure 2:**
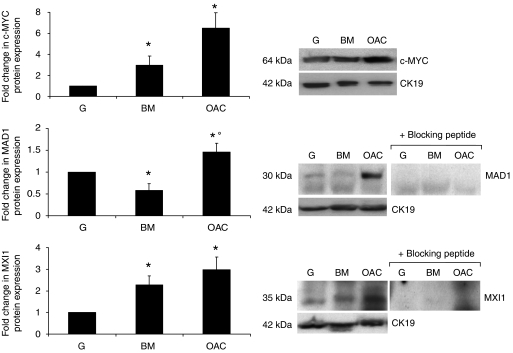
MYC/MAX/MAD network protein expression in Barrett's metaplasia and oesophageal adenocarcinoma. Expression of c-MYC, MAD1 and MXI1 protein was examined in Barrett's metaplasia (BM *n*=6) and oesophageal adenocarcinoma (OAC *n*=15) with matched normal gastric mucosa (G) by western blotting. Immunoreactive bands were assessed by semi-quantitative densitometry. Expression in BM and OAC is expressed relative to G (normalised to one); cytokeratin 19 (CK19) was employed for normalisation of protein loading. A representative western blot for each protein is shown alongside densitometry data representing mean expression change ±1 s.e.m. ^*^ denotes significant change relative to G, ° denotes significant change between BM and OAC.

Western blotting analysis was employed to confirm the alteration in expression at the protein level. In comparison to matched normal gastric controls the expression of c-MYC and MXI1 was significantly upregulated in Barrett's metaplasia; conversely, the expression of MAD1 was significantly lower in the metaplastic lesion than in the normal mucosa (Figure 2). In accordance with the mRNA data c-MYC, MAD1 and MXI1 expression was significantly higher in oesophageal adenocarcinoma than in matched normal gastric controls. While a difference in *MYC* and *MXI1* was demonstrated between Barrett's metaplasia and adenocarcinoma at the level of mRNA, there was no significant alteration in protein expression in malignancy. However, while *MXD1* expression was not altered at the transcript level, MAD1 protein was expressed more highly in adenocarcinoma than Barrett's metaplasia.

### Immunolocalisation of MYC/MAX/MAD network proteins in the progression to oesophageal adenocarcinoma

**Figure 3:**
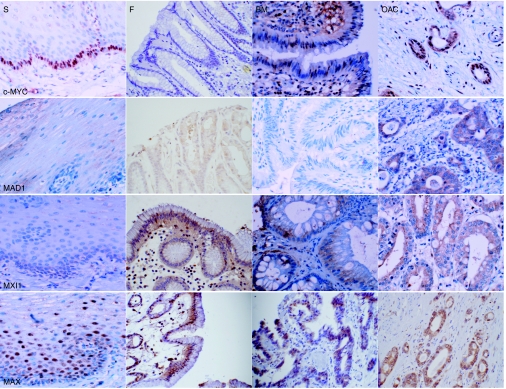
Immunolocalisation of MYC/MAX/MAD network proteins in Barrett's metaplasia and oesophageal adenocarcinoma. Paraffin sections of normal oesophagus, normal gastric fundus, Barrett's metaplasia and oesophageal adenocarcinoma were subjected to immunohistochemistry using antibodies designed against c-MYC, MAD1, MXI1 and MAX. Magnification × 40.

Immunohistochemical staining was utilised to establish MYC/MAX/MAD network protein localisation in normal oesophageal and gastric epithelia, Barrett's metaplasia, dysplastic Barrett's epithelium and oesophageal adenocarcinoma (Figure 3). To allow semi-quantitative evaluation of protein expression, the epithelial component of each section was scored as described in the methods for intensity of immunoreactivity and percentage of epithelial cells stained (Di Martino *et al*, 2006) (Table 2).

c-MYC staining in native squamous oesophageal epithelium was confined to the nuclei of cells in the proliferative basal layer. No immunoreactivity was observed on sections of normal gastric fundus. Barrett's metaplasia exhibited weak to moderate nuclear staining; however, heterogeneity is suggested since staining was not evident in all glands within a single specimen. Dysplastic Barrett's glands demonstrated evidence of nuclear and cytoplasmic c-MYC expression. Immunoreactivity was widespread and intense in the majority of adenocarcinoma sections, indicating both nuclear and cytoplasmic expression in most cases (Figure 3). Semi-quantitative analysis suggested that c-MYC expression in dysplastic Barrett's mucosa and adenocarcinoma tissue was significantly higher than in normal oesophageal and gastric mucosae. Expression in Barrett's metaplasia was lower than in both dysplasia and adenocarcinoma but significantly higher than in normal stomach (Table 2).

Weak diffuse cytoplasmic MAD1 immunoreactivity was observed in normal oesophageal and gastric mucosae and Barrett's metaplasia. In squamous epithelium staining was localised to the suprabasal differentiated compartment of the epithelium, gastric mucosa also demonstrated evidence of nuclear immunoreactivity in some of the positively stained glands. Only 25% of Barrett's metaplasia displayed MAD1 immunoreactivity, similarly half of the examples of dysplastic Barrett's epithelium remained negative; immunoreactivity on the positive sections was indicative of increased cytoplasmic expression in both cases. Staining in adenocarcinoma was more evident, with sections demonstrating cytoplasmic staining with intensity ranging from weak to strong (Figure 3). Semi-quantitative analysis indicated that expression in adenocarcinoma was greater than normal oesophageal and gastric epithelia and non-dysplastic Barrett's metaplasia (Table 2).

The majority of normal oesophageal epithelium sections did not display any MXI1 immunoreactivity, in those that did staining was weak and localised to the nuclei and cytoplasm of the epibasal cells. In gastric fundus expression of MXI1 was limited, with a small amount of weak cytoplasmic staining in some sections; the nuclei of the adjacent lymphoid cells stained positively for MXI1. Immunoreactivity was evident in approximately one-third of Barrett's metaplasia specimens and demonstrated cytoplasmic expression of weak to moderate intensity. A pattern of expression also evident in dysplastic tissue with staining consistently moderate in intensity. MXI1 immunoreactivity in oesophageal adenocarcinomas was largely moderate in intensity and cytoplasmic in localisation (Figure 3). Epithelial immunoreactivity in tumours was significantly more intense and widespread than in normal oesophagus, stomach and benign and dysplastic Barrett's metaplasia (Table 2).

In normal oesophageal epithelium MAX immunoreactivity was moderately intense in the nuclei and weak in the cytoplasm of the suprabasal layers of the stratified epithelium. In normal fundal mucosa immunoreactivity was moderately intense and largely nuclear; Barrett's metaplasia however, displayed weak immunoreactivity that was predominantly cytoplasmic. Staining in Barrett's dysplasia was also cytoplasmic but of moderate intensity. Adenocarcinoma sections displayed MAX immunoreactivity localised to both nuclei and cytoplasm (Figure 3). Semi-quantitative analysis suggested that MAX expression was significantly higher in adenocarcinoma than in normal mucosae and Barrett's metaplasia (Table 2).

### Overexpression of c-MYCER and MAD1 in SEG1 cells

**Figure 4:**
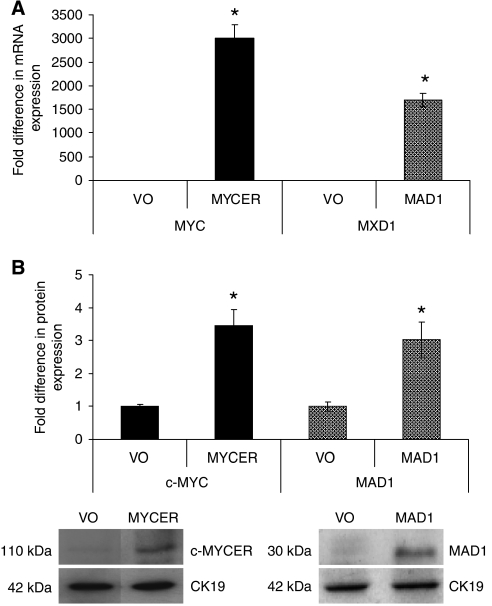
Confirmation of expression in transfected SEG1 cells. SEG1 cells were transfected with pcDNA3.1/Zeo-MYCER, pcDNA3-MAD1 or the corresponding empty vector (VO); in the case of MYCER the chimeric product was then activated by the addition of 4-OHT. (**A**) qRT–PCR was utilised to assess *MYC* (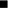) or *MXD1* (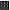) mRNA expression. (**B**) Western blotting demonstrated the expression the chimeric protein in SEG1-MYCER or MAD1 in SEG1-MAD1. Densitometric scanning approximated the fold increase in expression; a representative blot is also shown. Values represent the mean of two experiments each performed in triplicate ±1 s.e.m. ^*^ denotes statistical significance (*P*<0.05).

An oesophageal adenocarcinoma cell line, SEG1, was transiently transfected with pcDNA3.1-MYCER, pcDNA3-MAD1 or the corresponding empty vectors. To activate c-MYC 4-OHT was applied to the cells 24 h after transfection. Significant overexpression of c-MYC and MAD1 was demonstrated at mRNA and protein level by qRT–PCR and western blotting, respectively 48 h following transfection (Figure 4A and B).

**Figure 5:**
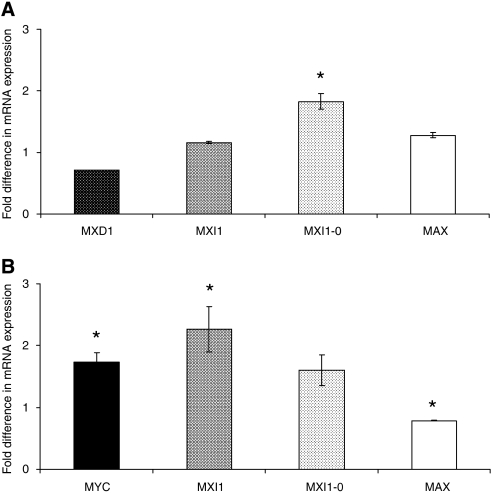
c-MYC network expression in SEG1 cells expressing exogenous MYC/MAX/MAD network proteins. (**A**) qRT–PCR was utilised to evaluate the expression of *MXD1* 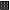, *MXI1* 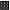, *MXI-0* 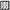 and *MAX* 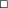 mRNA in SEG1 cells transiently overexpressing MYCER. Relative gene expression is expressed as a ratio of SEG1-MYCER not stimulated using 4OHT normalised to one. (**B**) Expression of *MYC* 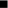, *MXI1* 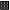, *MXI-0* 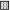 and *MAX* 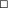 mRNA was assessed in SEG1 cells transiently overexpressing MAD1. Relative gene expression is expressed as a ratio of mock transfected cells normalised to one. Data represent the mean of two independent experiments each performed in triplicate ±1 s.e.m. ^*^ denotes statistical significance (*P*<0.05).

The expression of other members of the MYC/MAX/MAD network was assessed in transfected cells. MYCER expressing cells demonstrated elevated levels of the mRNA encoding MXI1-0 (*P*<0.001) (Figure 5A), whereas MAD1-overexpressing SEG1 cells demonstrated elevated *MYC*, *MXI1* and repressed *MAX* expression (*P*=0.002, *P*=0.014, *P*=0.03, respectively) (Figure 5B). Activated SEG1-MYCER cells demonstrated significantly higher BrdU incorporation over 72 h than the untreated controls (107±2%, *P*=0.024), conversely SEG1-MAD1 proliferated at a significantly slower rate than mock transfected controls (87±2%, *P*=0.006). These overexpression studies were similarly reproduced in other cell lineages including the oesophageal cell line OE21 (data not shown).

## DISCUSSION

Progressive overexpression of *MYC* in the oesophageal metaplasia-dysplasia-adenocarcinoma sequence has been observed previously (Tselepis *et al*, 2003; Schmidt *et al*, 2007); prior to this study however, the expression of other members of the MYC/MAX/MAD network had not been studied in any detail in the oesophagus. In the only study in this area the gene encoding MAD1 was identified as one of six genes downregulated at the mRNA level in oesophageal adenocarcinoma when compared with normal squamous oesophageal mucosa (Hourihan *et al*, 2003). Expression of c-MYC and MAD in normal oesophageal epithelium was consistent with previous studies in tissues compartmentalised with respect to proliferation and differentiation (Larsson *et al*, 1994; Chin *et al*, 1995; Hurlin *et al*, 1995a). c-MYC was confined to the proliferative compartment, the basal cell layer, a pattern previously observed in epidermis and colonic epithelium (Osterland *et al*, 1990; Tselepis *et al*, 2003). MAD1 expression was evident in the suprabasal layers, in differentiating postmitotic cells consistent with the role of MAD1 in terminal differentiation (Vastrik *et al*, 1995; Lymboussaki *et al*, 1996; Queva *et al*, 1998).

In light of the observations of reduced MAD1 expression in Barrett's metaplasia and previous evidence of *MXD1* repression in oesophageal adenocarcinoma (Hourihan *et al*, 2003), the trend towards upregulation of MAD1 in adenocarcinomas was not anticipated and would appear to be in conflict with the suggestion that MAD1 may have a role as a tumour suppressor (Ayer *et al*, 1993; Zervos *et al*, 1993; Cultraro *et al*, 1997; Queva *et al*, 1998). In support of the observations made here, MAD1 expression has been identified in benign and malignant murine skin tumours where the differentiation capacity was retained (Lymboussaki *et al*, 1996). Similarly, MAD1 expression has been identified in invasive ductal breast carcinomas of a well-differentiated phenotype (Han *et al*, 2000). It is possible therefore that an increase in MAD expression could be attributed to a negative feedback pathway to counteract aberrant MYC signalling and may be consistent with a role as a tumour suppressor. Indeed MXI1 upregulation has previously been observed downstream of MYC (Schuhmacher *et al*, 2001).

The overexpression of MXI1 in tumours may also, in part, be explained by the presence of differentially transcribed isoforms of MXI1 that lack important functional domains (Engstrom *et al*, 2004; Kawamata *et al*, 2005). MXI1-0 possesses an alternative first exon and as it does not express the same Sin3-interaction domain (SID), it is not capable of suppressing MYC-induced transformation. Overexpression of this isoform has been reported in glioblastomas in comparison with normal brain tissue (Engstrom *et al*, 2004) and therefore may represent the overexpressed isoform in oesophageal adenocarcinomas. Analysis of transcript levels by qRT–PCR allowed the expression of these isoforms to be differentiated; our data indicate that both isoforms are involved in the observed MXI1 overexpression in tumours, but that the putatively dominant negative isoform may be involved in the evolution of Barrett's metaplasia. Haematological tissue expresses three additional MXI1 isoforms, lacking either the SID-encoding first exon, the DNA-binding domain encoded by exon 3 or both these domains, which could also be considered in the oesophagus. Like MXI1-0, these isoforms may potentially exert a dominant negative effect (Kawamata *et al*, 2005).

MAX expression is widely acknowledged to be ubiquitous and in excess of other network members, whose expression is more tightly regulated (Blackwood and Eisenman, 1991; Berberich *et al*, 1992). Therefore, it was interesting to observe an increase in its expression in oesophageal adenocarcinoma. It has been suggested that although MAX is likely to be in excess of c-MYC and other binding partners in most circumstances, it may be limiting during the period when c-MYC levels are sharply elevated during cell cycle entry (Walker *et al*, 2005). This raises the possibility that MAX may also be limiting in tumours where c-MYC levels are very high. The observed overexpression demonstrated here may act to overcome this limitation, and may limit the antagonistic relationship between c-MYC and the MAD proteins that are evidently coexpressed. Increased MAX expression has been linked to increased proliferation (Martel *et al*, 1995), but the mechanism by which MAX overexpression may occur in tumours is elusive.

Cytoplasmic expression of MYC/MAX/MAD network proteins may be an indication that, although overexpression occurs, these proteins may not be completely functional. It has been suggested that cytoplasmic localisation of c-MYC in colon cancer may be due to alterations in the C terminus of the protein, reducing the efficiency of nuclear targeting (Royds *et al*, 1992). While this may also be the case for other network members, evidence of murine mmip2-mediated cytoplasmic translocation of mad proteins (Yin *et al*, 1999) and cytoplasmic localisation of MXI-0 (Engstrom *et al*, 2004) lend other potential mechanisms to cytoplasmic expression.

Consistent with *in vitro* and transgenic models of *MYC* amplification (Pelengaris *et al*, 2002), an increase in cellular proliferation was demonstrated following c-MYCER activation in SEG1 cells. c-MYC overexpression resulted in an increase in the expression of *MXI1-0* but had no effect on MXI1 suggesting alternative factors involved in their expression. Indeed Engstrom *et al* (2004) suggest that regulation of *MXI1-0* may differ from the AP2-mediated repression of the *MXI1* promoter (Benson *et al*, 1999). As MXI1-0 is thought to lack the antagonistic effects of MXI1, one may suggest that increased expression may facilitate the activity of c-MYC.

MAD1 overexpression in SEG1 cells resulted in a reduction in cellular proliferation at 72 h in concordance with earlier studies associating MAD1 with reduced cell cycling and compromised tumourigenicity and colony formation (Chen *et al*, 1995; Wechsler *et al*, 1997). MAD1 overexpression has previously been associated with accumulation of cells in G0/G1 mediated in part by limited G1 phase cyclin/CDK complex kinase activity and moderate increases in the expression of CDK inhibitors p27^KIP1^ and p21^CIP1^. Although the observations made in SEG1 cells are consistent with previous overexpression studies, they oppose the observation that MAD1 is overexpressed in oesophageal adenocarcinoma.

To conclude, the overexpression of c-MYC in Barrett's metaplasia and oesophageal adenocarcinoma has been confirmed. Interestingly, this was accompanied by an overexpression of c-MYC antagonists MAD1 and MXI1 in many tumours. These observations demonstrate that the expression patterns and regulation of this network of proteins may be more complex than initially predicted. This may, in part, be due to the natural heterogeneity of tumour tissue, indeed localisation by immunohistochemistry demonstrated heterogeneous staining. Multiple isoforms of MXI1 have been identified in a variety of tissues, which raises the possibility that alternative isoforms of other network members might exist that interfere with their previously known functions. Therefore, it is worth considering that any MYC-targeted therapy approach may also need to take into account the action of the MAD family proteins.

## Figures and Tables

**Table 1:** Taqman probe and primer sequences used for qRT–PCR

**Gene**	**Sequence**
*MYC*	
Forward	TCAAGAGGTGCCACGTCTCC
Reverse	TCTTGGCAGCAGGATAGTCCTT
Probe	CAGCACAACTACGCAGCGCCTCC
	
*MXD1*	
Forward	CCTTAAAACGGAGGAACAAATCC
Reverse	AGCGAAGATGAGCCCGTCTA
Probe	AAAAGAATAACAGCAGTAGCAGATCAACTCACAATGAAA
	
*MXI1 (Exon 1/2 boundary)*	
Forward	GGGAGCGAGAGTGTGAACATG
Reverse	TTCTGTGCCCGGCTCAAC
Probe	CCCGACTGCAGCATTCAAAGCCC
	
*MXI1-0 (Exon 0/2 boundary)*	
Forward	CTACCTGGAGCAGATCGAGAAAG
Reverse	TCGGCATGGACGGGAAT
Probe	AAACAAAAAGTGTGAACATGGCTACGCCTC
	
*MAX*	
Forward	AGGTGGAGAGCGACGAAGAG
Reverse	GTGCATTATGATGAGCCCGTTT
Probe	CCGAGGTTTCAATCTGCGGCTGAC

**Table 2:** Semi-quantitative analysis of c-MYC network proteins in the oesophagus

**Protein**	**Mean score of immunoreactivity**				
	**S**	**F**	**BM**	**BD**	**OAC**
c-MYC	1.50±0.2	0.50±0.3	**2.42±0.5^‡^**	4.00±0.7*^‡^°	7.70±0.8*^‡^°
MAD1	1.00±0.4	1.25±0.3	0.54±0.3	1.81±0.5	3.97±0.6*^‡^°
MXI1	0.50±0.3	1.50±0.4	0.78±0.2	1.42±0.6	4.06±0.5*^‡^°
MAX	4.33±1.3	2.00±1.2	1.90±0.3	**3.7±0.77°**	8.00±0.69*^‡^°

Immunohistochemistry for c-MYC, MAD1, MXI1 and MAX was performed on paraffin sections of normal squamous oesophageal mucosa (S), normal gastric fundus (F), Barrett's metaplasia (BM), dysplastic Barrett's (BD) and oesophageal adenocarcinoma (OAC). Immunoreactivity was scored as described in Materials and methods. The mean score is displayed ±1 s.e.m. ^*^ denotes significant change in comparison to S; **^‡^**denotes statistical significance compared to F; ° denotes significance relative to BM, (*P*<0.05).
